# Secondary neurodegeneration following Stroke: what can blood biomarkers tell us?

**DOI:** 10.3389/fneur.2023.1198216

**Published:** 2023-09-01

**Authors:** Stefano Brunelli, Emilia Giannella, Mirko Bizzaglia, Domenico De Angelis, Giulia Maria Sancesario

**Affiliations:** ^1^NeuroRehabilitation Unit 4, IRCCS Santa Lucia Foundation, Rome, Italy; ^2^Clinical Neurochemistry Unit and Biobank, IRCCS Santa Lucia Foundation, Rome, Italy; ^3^Radiology and Diagnostic Imaging Unit, IRCCS Santa Lucia Foundation, Rome, Italy

**Keywords:** biomarkers, stroke, secondary neurodegeneration, neurofilaments light, rehabilitation, brain-derived neurotrophic factor (BDNF), neuron specific enolase (NSE), tau

## Abstract

Stroke is one of the leading causes of death and the primary source of disability in adults, resulting in neuronal necrosis of ischemic areas, and in possible secondary degeneration of regions surrounding or distant to the initial damaged area. Secondary neurodegeneration (SNDG) following stroke has been shown to have different pathogenetic origins including inflammation, neurovascular response and cytotoxicity, but can be associated also to regenerative processes. Aside from focal neuronal loss, ipsilateral and contralateral effects distal to the lesion site, disruptions of global functional connectivity and a transcallosal diaschisis have been reported in the chronic stages after stroke. Furthermore, SNDG can be observed in different areas not directly connected to the primary lesion, such as thalamus, hippocampus, amygdala, substantia nigra, corpus callosum, bilateral inferior fronto-occipital fasciculus and superior longitudinal fasciculus, which can be highlighted by neuroimaging techniques. Although the clinical relevance of SNDG following stroke has not been well understood, the identification of specific biomarkers that reflect the brain response to the damage, is of paramount importance to investigate *in vivo* the different phases of stroke. Actually, brain-derived markers, particularly neurofilament light chain, tau protein, S100b, in post-stroke patients have yielded promising results. This review focuses on cerebral morphological modifications occurring after a stroke, on associated cellular and molecular changes and on state-of-the-art of biomarkers in acute and chronic phase. Finally, we discuss new perspectives regarding the implementation of blood-based biomarkers in clinical practice to improve the rehabilitation approaches and post stroke recovery.

## 1. Introduction

Stroke is a major global health burden, affecting more than 12 million of individuals each year worldwide (1). According to the latest data, stroke is the second leading cause of death worldwide and third leading cause of disability (2). The economic impact of stroke is also significant, with direct and indirect costs estimated to be, in 2017, around 861 billion of international dollars each year, i.e., 1.12% of the global Gross Domestic Product (GDP) (1). Despite progress in understanding the underlying mechanisms of stroke and the development of new treatments, much work remains to be done to reduce the impact of stroke on individuals and society, not only during the acute phase but also during long-lasting invalidity in the following years.

The most common type of stroke, the ischemic one, occurs when a blood clot blocks the flow in an artery in the brain. The less frequent, the hemorrhagic stroke, can result from a ruptured blood vessel or from a structural abnormality of a blood vessel in the brain. Both stroke conditions cause local hypoxia and reduction of supply of other nutrients, with consequent damage or death of neurons (3, 4). The location, extensiveness and the number of the lesions can determine the degree of disability.

Clinically, the evaluation of stroke severity is performed through clinical measures by an experienced stroke clinician. Clinical assessments can provide valuable information about the severity of a stroke, but they are often imprecise and have only a moderate correlation with actual brain tissue damage and long-term outcomes. To determine the site of stroke area and the extent of brain damage the current strategies include standard neuroimaging such as conventional brain magnetic resonance (MRI) and computed tomography (CT) of the brain. There is also the possibility in the follow-up after the stroke, to study the evolution of the disease using advanced neuroimaging techniques such as functional MRI, perfusion imaging, diffusion imaging, magnetic resonance spectroscopy, and dual-energy computerized tomography.

Based on the area involved, different clinical phenotypes can be observed, with overlapping signs, including motor impairment, speech or cognitive deficits, as contralateral hemi-spatial neglect, memory or executive dysfunctions, visual field defects, dysphagia, urinary incontinence and other symptoms.

Furthermore, functional disturbances in remote parts of the brain, connected with the area with neuronal damage, were reported (5). This phenomenon could be explained by the secondary neurodegeneration (SNDG) that gradually spreads to different brain structures, even if not directly affected by reduction in cerebral blood flow caused by the initial stroke (6).

The progressive death of neurons, axonal degeneration and gliosis in distal regions of the brain have been observed in several experimental (7–9) and in clinical neuroimaging studies (10–14). The site of the infarct significantly influences the spreading of secondary changes (10), and the progressive involvement of these areas can lead to a further worsening of deficits and disability.

Actually, investigating the effect of SNDG following stroke on patients’ outcome, disability and recovery has reached increasing interest. Evidence suggests that measurement of circulating brain derived biomarkers in cerebrospinal fluid (CSF) or blood are a useful tool to evaluate the pathophysiological and biochemical modifications occurring during pathological processes (15, 16). Here, we describe: (i) the most frequent structural cerebral modifications occurring after a stroke; (ii) associated cellular and molecular changes; (iii) state-of-the-art and advances in the field of biomarkers in acute phase and in secondary neurodegeneration. In particular, we focus on markers of neuronal and astroglial damage, namely neurofilament light chain (Nf-L), tau proteins, neuron-specific enolase (NSE), S100 calcium binding protein B (S100b), as well as on marker of regeneration, as brain-derived neurotrophic factor (BDNF). Finally, we discuss new perspectives regarding the implementation of blood-based biomarkers in clinical practice to improve therapeutic intervention and neurorehabilitation.

### 1.1. Pathophysiology of Stroke and secondary neurodegeneration

**Figure 1:**
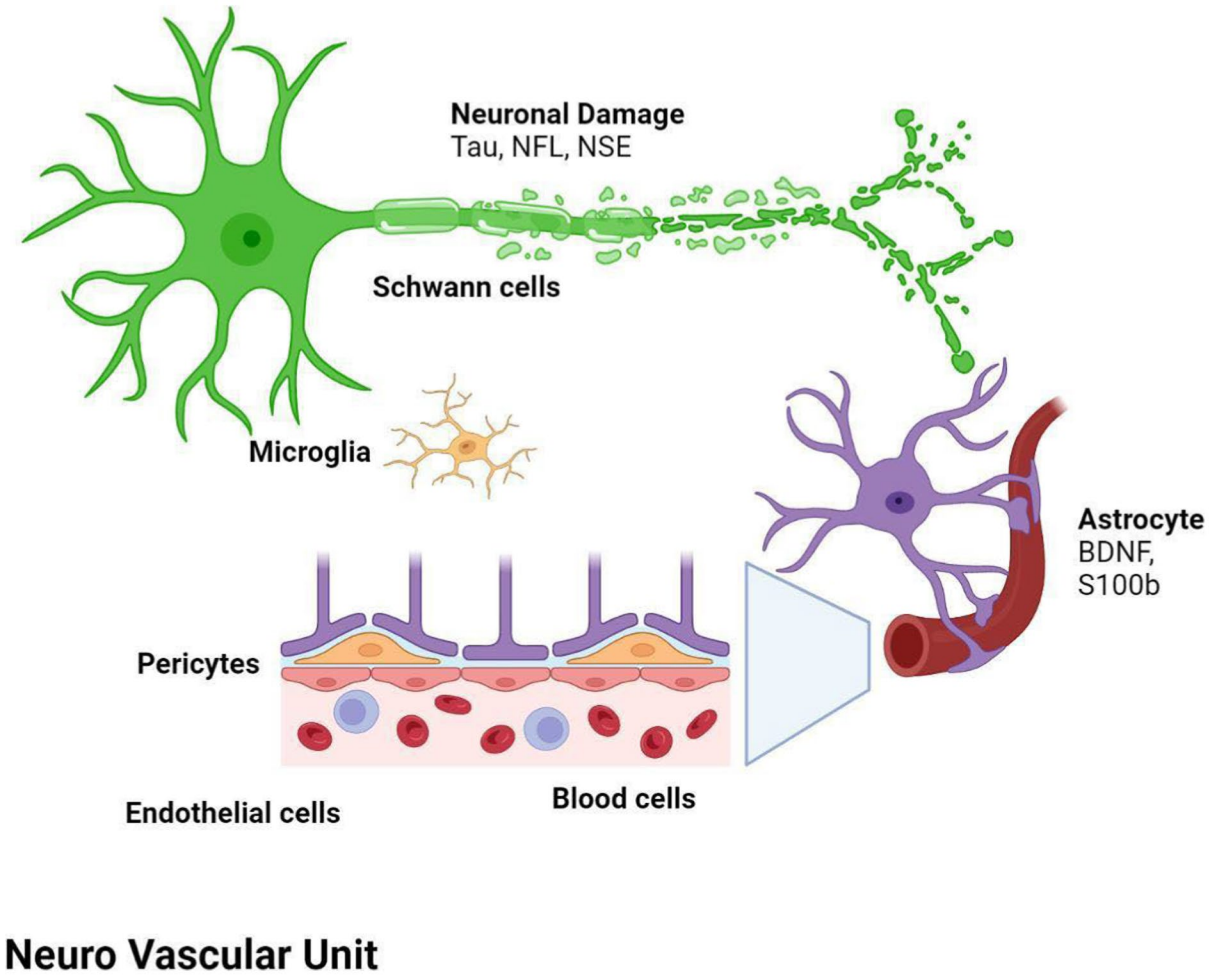
Both ischemic and hemorragic stroke cause local hypoxia and reduction of supply of other nutrients, with consequent damage or death of neurons and multiple cell types, glial, astrocytes, pericytes, smooth muscle cells, endothelial cells, oligodendrocytes, microglia and neural and glial precursor cells, which together constitute the neurovascular unit (NVU). Biomarker changes in CSF and blood reflect the extent of brain injury and development of pathological changes, and correlate with severity of damage and the activation of reparative mechanisms. BDNF, brain-derived neurotrophic factor; NF-L, neurofilament light proteins; NSE, neuron specific enolase; S100b, S100 calcium-binding protein B.

After stroke, a cascade of inflammatory and degenerative mechanisms is triggered that ultimately determine the extent and severity of damage, the development of late secondary neurodegeneration, and the degree of disability or recovery (17). In the first phase, injury of the central nervous system (CNS) results from the dysfunction and death of neurons and of multiple cell types, including astrocytes, pericytes, smooth muscle cells, endothelial cells, oligodendrocytes, microglia and neural and glial precursor cells, which together constitute the so-called neurovascular unit (NVU) (18, 19). The NVU has been hypothesized to regulate the balance of the neural–glial–vascular signaling in a biphasic mode, that is mainly oriented to the damage during the acute injury phase and to the regeneration in the chronic recovery (20–22). Thus, post-stroke recovery results from reversal of tissue dysfunction, by promoting neurogenesis, angiogenesis and vascular remodeling (23–27) (Figure 1).

The imbalance between persistence of chronic inflammatory degenerative status and neurogenesis is considered responsible for persistent neurological dysfunction and impairment of functional recovery following damage of the CNS (28–30). Indeed, it is known that the occurrence of brain lesions, due to trauma or stroke injury, increases the incidence of late impairments and chronic neurodegenerative conditions, such as Alzheimer’s disease (AD), Parkinson’s disease (PD) and chronic traumatic encephalopathy (31, 32).

SNDG refers to neurophysiological and histopathologic changes occurring in non-ischemic remote brain regions that have anterograde or retrograde synaptic connections with the primary lesion site (6); thus, SNDG has been hypothesized to be a potential modulator of post-stroke functional disorders (33).

Most of the knowledge on neurodegenerative processes associated with stroke is based on studies with neuroimaging and in particular brain MRI, providing an excellent anatomical detail and gray/white matter contrast. For this reason, structural MRI, using conventional sequences, especially T1, T2-weighted, in addition to contrast enhancement, has become the accepted standard for routine examination of the brain, offering high sensitivity to anatomical location and morphological characteristics of pathological processes.

Studying the SNDG with conventional MRI, disruption to global functional connectivity has been revealed in the ipsi-lesional corticospinal tract and in the inter-hemispheric connections (corpus callosum), in the bilateral inferior fronto-occipital fasciculus and in the bilateral superior longitudinal fasciculus (34–36), in the ipsilateral thalamus, in the substantia nigra, hippocampus and in amygdala (37–39).

The involvement of these areas distal to the primary lesion defines the onset of cognitive and behavioral symptoms different from those primarily related to stroke area (36, 40). For example, involvement of the thalamus can lead to hyperalgesia; damage in the substantia nigra provokes Parkinson-like symptoms (slow movements, tremor, stiffness and difficulty with walking and balance); involvement of amygdala results in difficulty with memory processing and emotional reactions, whereas an involvement of hippocampus to memory impairment (Table 1).

**Table 1:** Brain structures involved in secondary neurodegeneration after stroke and MRI: localization, functions, and clinical signs.

Brain area	Functions	Clinical signs	
Corticospinal tract (mainly ipsilateral)	Major pathway involved in voluntary motor activity	Paralysis, increased muscle tone, hyperactive deep muscle reflexes (41)	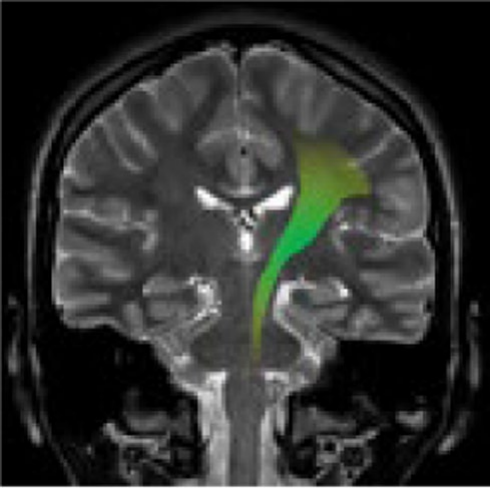
Corpus callosum	The white matter structure that permits the communication between the right and left sides of the brain	Cognitive and behavioral disability, agraphia, apraxia, tactile anomia, alien limb syndrome (42)	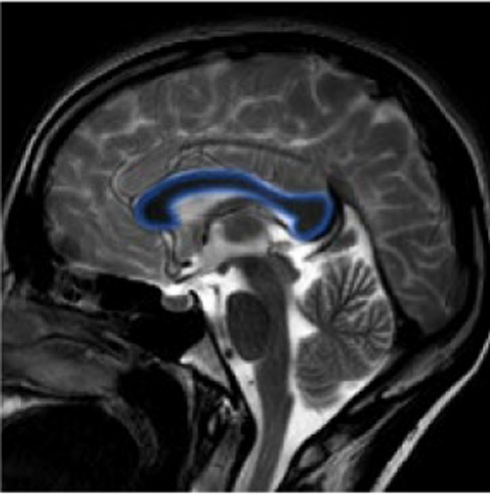
Inferior fronto-occipital fasciculus (bilateral)	The associative fibers that bridge frontal, parietal and occipital lobes	Involvement of object, face and place processing, reading, lexical and semantic processing, emotion processing, and visual memory (43)	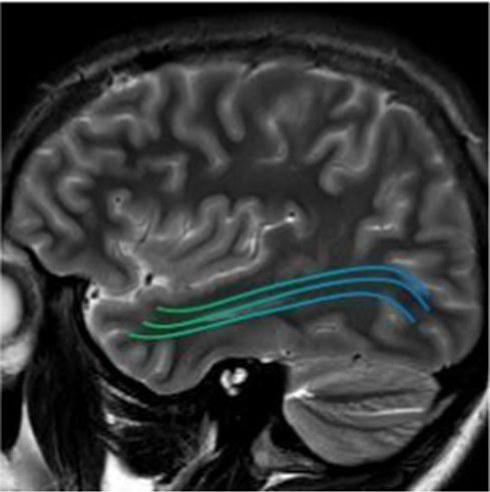
Superior longitudinal fasciculus (mainly bilateral)	The associative fiber bundle that connects frontal, temporal, and parietal lobes	Deficit in speech processing or visuospatial functioning (44)	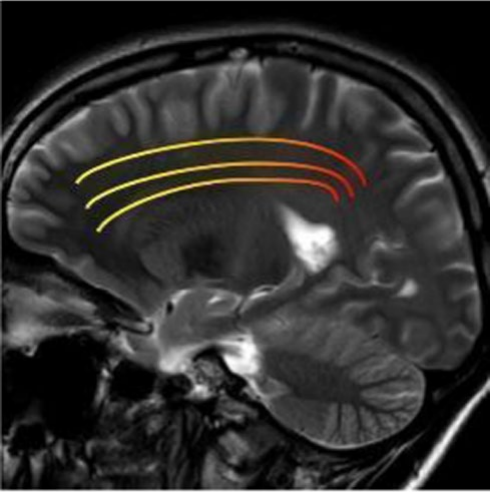
Thalamus (ipsilateral)	The deep, mostly gray matter, structure involved in sensory and motor signals, regulation of consciousness and alertness, cognitive functions and pain regulation	Deficit in executive functions, memory, emotion, sleep–wake cycle, hyperalgesia, sensory impairment (45)	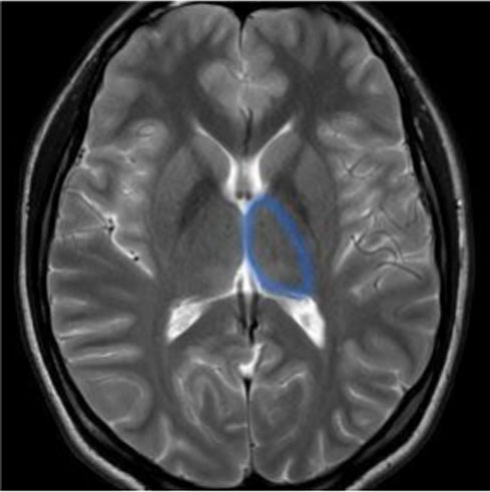
Substantia nigra (ipsilateral)	The gray matter structure of the midbrain important for motor and movement control and some cognitive functions, such as learning, reward, emotions	Slow movements, tremor, rigidity, imbalance (Parkinson-like symptoms), involvement of regulation of emotion, and motivation (46)	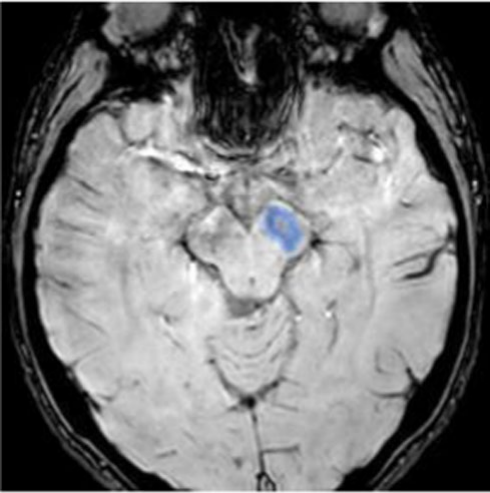
Hippocampus	The brain structure located in medial part of each temporal lobe crucial for memory and learning functions	Cognitive dysfunctions, depression, epilepsy (47)	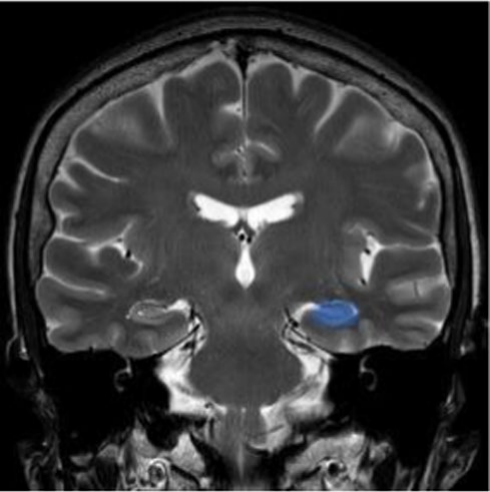
Amygdala	The cerebral nuclei located deep in the temporal lobes acting as the center of behavior, for memory and learning functions	Memory deficits, anxiety, behavioral changes, autonomic dysfunctions (48)	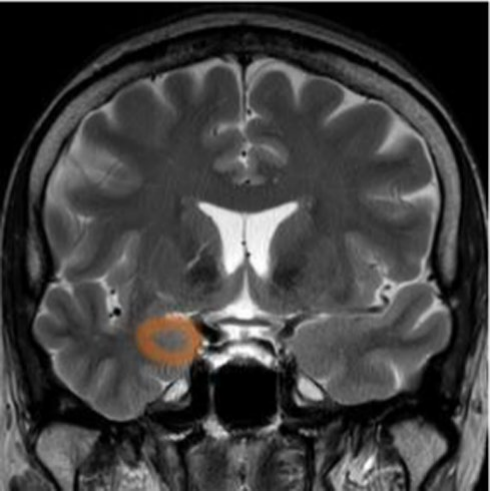

After stroke, impairment in brain structures not directly involved in the primary lesion may occur. This phenomenon could be explained by the secondary neurodegeneration (SNDG) that gradually spreads to different brain structures, even if not directly affected by reduction in cerebral blood flow caused by the initial stroke. Based on the area involved, different clinical phenotypes can be observed, with overlapping signs, including motor impairment, speech or cognitive deficits, as contralateral hemispatial neglect, memory or executive dysfunctions, visual field defects, dysphagia, urinary incontinence and other symptoms.

In addition, other pathogenic mechanisms occurring after stroke have been reported as potential factors leading to cognitive decline. Interestingly, some studies described alterations of functional connectivity measured with functional MRI in different parts of cortex that appear structurally normal, not contiguous to the stroke area (49, 50). This suggests that uninvolved areas of the brain, even if structurally normal, may have functional impairment. It was supposed that the stroke lesion can disrupt the mutual balanced inhibition between hemispheres by decreasing the inhibition of the contralateral non lesioned hemisphere and by reducing the number of cortical-spinal tract projections to the other brain areas, with the effect of a remote functional depression (51).

Following ischemic stroke, microglia and astrocytes are activated within hours, inducing the production of cytokines and chemokines and the infiltration of leukocytes (52, 53). Oxidative stress, a disturbance in the balance between the production of reactive oxygen species and antioxidant defenses, is induced in cerebral ischemia especially through inflammation and reperfusion, increasing the production of free radicals (54). After the activation of the peripheral immune response, macrophages and neutrophils are released by the spleen into the bloodstream, which can easily reach the brain due to the alteration of the blood–brain barrier (55). This immune response, associated with the activation of resident inflammatory cells such as microglia and astrocytes, has been demonstrated to contribute to the development of SNDG (56, 57).

On a cellular level, stroke lesions cause the disruption of the structure of the neuron’s cytoskeleton (58). Those changes in cytoskeletal structure and subsequent neuron instability and final neuronal death are associated with the formation of protein aggregates and the release of cytoplasmic proteins in the extracellular space, representing a valuable surrogate index of acute necrosis or slow

neurodegeneration (59, 60).

Among these, tau proteins, a microtubule associated protein, and the corresponding hyperphosphorylated forms, p-tau, are well-established biomarkers of neurodegeneration, and represent predictors of functional outcome (61) or development of cognitive dysfunction after cerebral ischemia (62–64). Further, increase of microtubule-associated protein (MAP2) reflects the fragmentation of neuronal dendrites, not only in the areas of stroke but even in other brain regions resulting in a widespread loss of synaptic plasticity (65, 66). Finally, stroke can also lead to accumulation of amyloid-beta (AB), a peptide that is the main component of amyloid plaques in the brains of subjects with Alzheimer’s disease (67).

The deposition of the neurotoxic aggregates of protein amyloid-beta could be further stimulated by the glial activation and pro-inflammatory cytokine release after cerebral ischemia, which persists for a long time and in some cases could determine the latter onset of Alzheimer’s type dementia (17).

## 2. Biomarkers of brain damage and Stroke

Brain atrophy patterns are recognized as signatures of neurodegenerative conditions, and have been included as topographical markers for AD and a number of other neurodegenerative diseases (68). Furthermore, regional atrophy rates have been shown to correlate with CSF and blood biomarkers of neurodegeneration. However, although the conventional neuroimaging techniques still represent the benchmark diagnostic tool, they may not have sufficient resolution to detect early changes in the brain at the cellular and molecular levels after stroke. Advanced neuroimaging may provide a better option to identify and follow up the biological processes involved in SNDG but not represent a real option because of the requirement of sophisticated and expensive instruments and trained personnel (69). Moreover, serial repetition of the neuroimaging is not feasible within a short interval to capture the evolution of the processes linked to SNDG. There is also to consider some general drawbacks of MRI such as long acquisition time, the possibility of movement artifacts, the contraindications in some patients with metallic surgical implants and patients’ claustrophobia.

Instead, blood-based biomarkers can reflect molecular and biochemical state in both normal and pathophysiologic processes, including neuronal and vascular injury, inflammation, oxidative stress, glial activation, etc. (70). Since stroke may induce blood brain barrier dysfunction (71), a progressive cross of brain-derived proteins into the bloodstream, and vice versa, may induce changes in concentration of several molecules and, accordingly, affect their clinical value. However, a great challenge is to understand whether assessment of biological markers after months or years after stroke, may be useful to early detect the occurrence of degenerative processes secondary to stroke.

The current knowledge on neurological biomarkers in stroke mainly takes advantage of findings in other neurodegenerative diseases, namely Alzheimer’s Disease (AD), Parkinson Disease (PD), dementia with Lewy bodies (LBD) and others. CSF biomarkers, including amyloid Beta 42 (Aβ42) and Aβ40, total tau (T-tau), phosphorylated tau (P-tau) have an added value in the differential diagnosis of AD and related disorders, including mixed pathologies, atypical presentations, and in case of ambiguous clinical dementia diagnosis (72–74).

**Figure 2:**
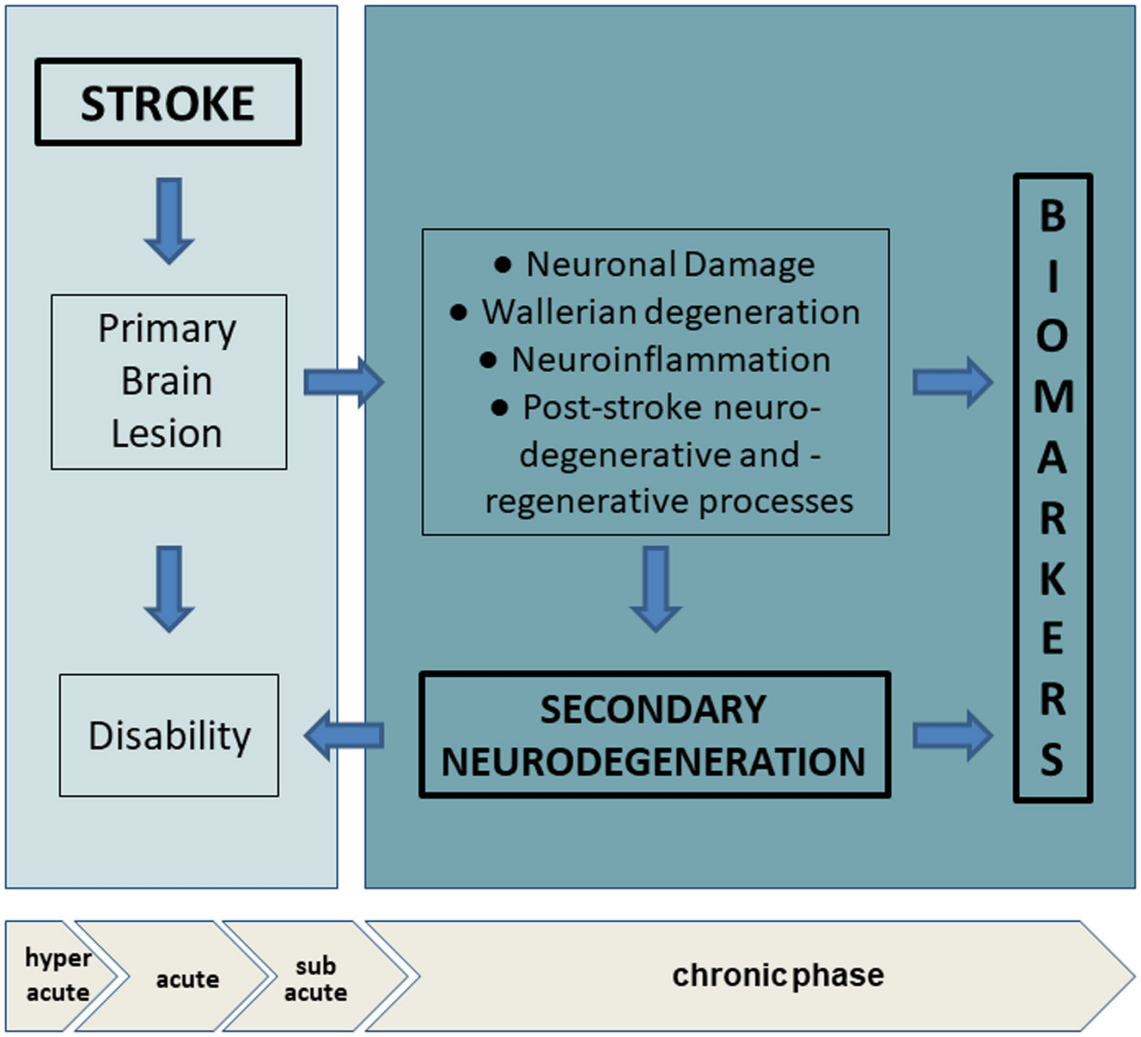
Biomarkers in chronic phase after stroke and secondary neurodegeneration. In hyperacute and acute phase, stroke provoques a primary brain lesion with consequent activation of a cascade of vascular, oxidative, inflammatory events. The location, extensiveness and the amount of the lesion can determine the degree of disability. In the chronic phase, adaptive neural plasticity and regenerative processes try to counteract wallerian degeneration, oxidative stress and neuroinflammation. Analysis of circulating biomarkers allow the evaluation of clinical silent changes, non-detectable with imaging technologies. Hyperacute (< 6 h), acute (6 to 72 h), subacute (after 7 days), chronic (after ~6 weeks).

Actually, a considerable number of published works have demonstrated that the blood level of some neuronal or glial proteins particularly increase after stroke and correlate with clinical features, severity and outcome, representing promising markers to evaluate the extent of brain injury (Table 2). In particular, we can differentiate: (1) markers of neuronal damage, namely neurofilament light chain (Nf-L), tau proteins, neuron-specific enolase (NSE); marker of astroglial damage, the S100 calcium binding protein B (S100b); (2) marker of neuroregeneration, the brain-derived neurotrophic factor (BDNF). Thus, longitudinal assessment of such biomarkers in subjects may reveal the presence of secondary subclinical degenerative or regenerative processes, providing information complementary to findings from routine examinations and neuroimaging (Figure 2).

**Table 2:** Summary of CSF and blood biomarkers with potential diagnostic characteristics for SDNG after stroke.

Study design	Sample size	Diagnosis/significance	Results	Reference(s)
Neurofilaments light (NfL)				
Meta-analysis study	7 studies, total 1,346 patients	Predictive - prognostic	Patients with higher serum NF-L had increased risk of poor functional outcome during follow-up, compared with those with lower NF-L	(75)
Clinical study	cohort 1,694 patients	Predictive - prognostic	Patients who developed post-stroke cognitive impairment had significantly higher levels of NF-L within 48 h of stroke onset; levels of NF-L were negatively correlated with cognitive impairment (MOCA <26) at 90 days after stroke onset, and directly correlated with age, cerebral infarction volumes and NIHSS score	(76)
Clinical trial	30 healthy controls; 2 independent cohorts of patients (total 380)	Predictive - prognostic	2 independent cohorts of patients with IS: cohort 1: serial serum sampling at hospital arrival (*n* = 196), at days 2, 3, and 7 (*n* = 89), and up to 6 months post stroke; Cohort 2: standardized MRI at baseline and at 6 months post stroke, and with cross-sectional serum sampling at 6 months (*n* = 95). The best association among clinical outcome and serum NF-L is measured at 7 days or within 48 h after symptom onset.	(77, 78)
Review	–	Diagnostic/ predictive	In the acute phase, high blood NF-L reflects the extent of neuronal injury; increased blood levels of NfL in individuals who survived stroke for more than 10 months were shown to predict functional improvement in the late phase after stroke	(79)
Clinical study	49 non-traumatic cervical artery dissection	Prognostic	sNfL levels are positively associated with NIHSS; Higher sNfL levels are associated with unfavorable outcome at 3 months	(80)
TAU				
Clinical study	9 patients	Biological + neuroimaging	Correlation among the magnitude of CSF t-tau increase and the volume of tissue damage after stroke	(81)
Clinical study	995 subjects Mayo Clinic Study of Aging (MCSA)	Risk factor, prognostic marker	Plasma NfL and T-tau are increased in stroke patients. The combination of having both higher NfL and T-tau, compared to either alone, is strongly associated with lower memory, global cognitive decline, brain atrophy and a higher number of infarcts cross-sectionally.	(82)
Clinical study	56 patients	Longitudinal analysis; prognostic marker	plasma tau progressively increases reaching a maximum peak 7 days after event; tau correlates with severity, long-term outcome and infarct volume	(83)
Clinical study	66 consecutive patients	Longitudinal analysis with neuroimaging; prognostic marker	66 patients, serial blood samples at 3, 6, 12, 18, 24, 48, 72, 96, and 120 h after stroke onset. Tau protein concentrations continuously increase from admission onward. NSE and tau release are highly correlated with severity of neurological deficits and infarct volume	(84)
Pilot study	25 patients	Prognostic marker	Analysis of kinetics of tau revealed a bimodal elevation after day 2 and 4, probably due to the occurrence of early first necrosis and secondary delayed neuronal death.	(85)
Experimental study	–	Animal model of ischemia	In animal models, tau protein dysfunction following ischemia may trigger neurofibrillary tangle-like tauopathy and neurofibrillary tangles	(64, 86, 87)
S100b				
Clinical study	26 patients and 26 controls	Predictive value	26 patients with an acute infarction in the territory of the MCA at day 0 (within 12 h after onset of symptoms), day 1 (24 h after stroke onset), and days 2, 3, 4, 5, 7 or 8, and 10 after stroke. S100b correlates with the severity of post-traumatic neurological deficit as well as with the infarct volume after stroke	(88)
Clinical study	39 patients	Predictive value	Serum S100B level at hospital admission and 24, 48, 72, 96, 120, and 144 h after symptom onset. S100B at 48 and 72 h after stroke onset provide the highest predictive values with respect to functional outcome and infarct volume	(89)
Clinical study	44 patients	Longitudinal, Predictive/prognostic	S-100 protein and NSE on admission and on days 3, 4, 7, and 14 after infarction. Peak plasma levels of S-100 protein at day 2 correlates with infarct volume and clinical outcome (Glasgow Outcome Scale)	(90)
Clinical study	32 patients	Predictive/prognostic	Serum concentrations of S100b (from 6 h) and enolase (from 24 h) are associated with the outcome at 3 months with a maximum of correlation obtained for protein S100b at 48 h	(91)
Review	–	Diagnostic value	Changes in S100b concentration reflect the extent of astroglial damage and the enlargement of the ischemic core	(92)
Clinical study	51 patients	Diagnostic- prognostic value	Changes in S100b concentration reflect the formation of malignant edema and of blood–brain barrier damage	(93)
Clinical study	23 patients	Monitoring marker	S100b level are surrogate marker for successful clot lysis in hyperacute middle cerebral artery occlusion	(94)
Clinical study	171 patients	Monitoring marker	Levels of S100b (2 day after intervention) correlate with the recanalization following mechanical thrombectomy, being low in case of successful recanalization, and oppositely being increased in case of ineffective recanalization and in patients who developed infarcts despite recanalization	(95)
Neuron specific enolase (NSE)				
Clinical study	150 patients; 101 controls	Predictive/prognostic	At the time of admission, NSE level correlates with stroke severity and with the degree of disability. NSE is associated with neurological worsening after 7 days of event	(96)
Systematic Review	12 studies, total 597 patients	Predictive/prognostic	Serum NSE levels are higher in stroke patients compared with controls and correlate with volume of infarct	(97)
Clinical study	66 consecutive patients	Longitudinal analysis; prognostic marker	66 patients, serial venous blood samples at 3, 6, 12, 18, 24, 48, 72, 96, and 120 h after stroke onset. Increase of NSE as well as of tau was highly correlated with severity of neurological deficits, infarct volume and with the functional outcome at 3 months. NSE release was associated with the neurovascular status on admission	(84)
Experimental study	Rat model ischemia	Pathological marker	Plasma NSE increase following permanent or transient middle cerebral artery occlusion	(98)
Clinical study	58 patients	Longitudinal, Predictive/prognostic	Serum NSE increases 2–3 h after onset of first stroke, afterwards, NSE decreased, followed by a secondary increase until day 5; the secondary increase indicates further release of NSE, which probably reflects a secondary mechanism of brain damage, ongoing neuronal cell death, or persistent disturbance of the blood–brain barrier	(99)
Clinical study	1,086 patients	Longitudinal, Predictive/prognostic	NSE correlates with stroke severity and prognosis after 1 year of follow up (NIHSS score and modified Rankin Scale (mRS) score)	(100)
Clinical study	44 patients	Longitudinal, Predictive/prognostic	S-100 protein and NSE on admission and on days 3, 4, 7, and 14 after infarction. Peak plasma levels of S-100 protein at day 2 correlates with infarct volume and clinical outcome (Glasgow Outcome Scale)	(90)
BDNF				
Clinical study	491 patients; 513 controls	Predictive/prognostic	BDNF decreases after stroke are associated with poor long term functional outcome	(101)
Meta analysis	4 studies, total 499 patients	Predictive/prognostic	BDNF decreases after stroke are associated with the development of post stroke depression	(102)
Clinical study	3,440 Framingham Study participants	Risk factor	10 years follow up, low level of BDNF are associated with increased risk of stroke	(103)
Meta-analysis study	62 studies; Total 1856 subjects with stroke and *n* = 1,191 healthy controls	Prognostic; monitoring marker	Subjects with stroke have lower BDNF levels compared to healthy controls; no significant difference in the level of BDNF through time points post stroke. BDNF levels are lower in patients with depression compared to non-depressed subjects, and positively affected by performing physical training in the early but not in the long term	(104)
Preclinical studies	Rat model	Therapeutic target	BDNF reduces the size of the lesion and secondary death	(105)
Preclinical studies	Rat model	Therapeutic target	BDNF promotes synaptogenesis, neuronal plasticity and recovery post stroke	(106)
Systematic review	21 studies	Prognostic; monitoring marker	The increase of BDNF concentration in the cortex is related to motor learning after-stroke	(107)

### 2.1. Neurofilaments

Neurofilament proteins are components of the cytoskeleton of neurons, classified on their molecular weight in light (NF-L), middle (NF-M) and heavy (NF-H). As a subunit of neurofilament, serum NF-L has emerged in the last few years as the most promising biomarker of axonal injury and neurodegeneration, mainly in multiple sclerosis (MS), showing potential applications for both patient monitoring and for observational and interventional trials (78, 108).

Recently, numerous studies have been carried out on patients with stroke showing significant correlation with severity, according to National Institutes of Health Stroke Scale (NIHSS) upon admission (77, 78, 80, 109, 110) and after clinical outcome (111).

A meta-analysis on ischemic stroke or transient ischemic attack patients demonstrated that patients with higher serum NF-L, had increased risk of poor functional outcome during follow-up, compared with those with lower NF-L, strengthening the value of NF-L as predictive biomarker for ischemic stroke outcome (75). Furthermore, a study on a cohort of 1,694 patients with first-ever acute ischemic stroke investigated the correlation among NF-L and post-stroke cognitive impairment (PSCI). Among the cohort, 60.74% of patients developed cognitive impairment. Interestingly, authors found that the PSCI group exhibited significantly higher levels of NF-L within 48 h of stroke onset; levels of NF-L were negatively correlated with cognitive impairment defined by Montreal Cognitive Assessment (MOCA) (MOCA <26) at 90 days after stroke onset, and directly correlated with age, cerebral infarction volumes and NIHSS score (76).

Importantly, results from different studies showed a wide heterogeneity regarding the sampling time, reporting best association among clinical outcome and serum NF-L measured at 7 days or within 48 h after symptom onset (77, 78). Pending multicentric studies and large-scale validation, it is however suggested to perform the analysis of NF-L before the 7-day time point.

Converging evidence suggests that NF-L level has an added predictive value apart from stroke severity. This may be explained by the presence of SDNG outside the infarct area, such as white matter tracts connected to the infarct, which could contribute to the development of cognitive impairment after the stroke (76). A study in the acute and post-acute phase after stroke confirmed that high levels of NF-L are associated with poor clinical outcome, and later on, the concentration of NF-L positively correlates with the occurrence of SDNG, as assessed by MRI.

Surprisingly, increase of NF-L has shown to predict functional improvement in the late phase after stroke in patients who survived for more than 10 months. This could suggest that the kinetic of NF-L may reflect two concurrent but very distinct processes of NF-L release, namely neuroaxonal injury and synaptic damage, that are features of secondary neurodegeneration, and oppositely of late adaptive neural plasticity. Indeed, while in the acute phase high blood NF-L seem to reflect the extent of neuronal injury, in the late phase increase of NF-L may serve as a biomarker of adaptive neural plasticity and a positive predictor of functional improvement and, therefore, effectiveness of neurorehabilitation (79).

Thus, results from different studies suggest that at different time points, NF-L levels may be considered a measure of structural brain lesion, complementary to MRI (80) but in the late phase after stroke, the interpretation of elevated blood levels of NF-L should not be therefore limited to the extent of injury or neurodegeneration (79).

### 2.2. Tau

Tau, a microtubule-associated protein that regulates stability and dynamics of axons, is considered a well-recognized marker of neuronal degeneration by a huge amount of both clinical and preclinical studies. In the research framework of AD, CSF tau is one of the core markers of the so called “ATN system,” where “A” refers to the value of an amyloid β biomarker (amyloid PET or CSF Aβ42); “T,” the value of a tau pathology biomarker (CSF p-tau or tau PET); and “N,” a quantitative or topographic biomarker of neurodegeneration or neuronal injury (112, 113).

So far, the assessment of total tau level in the CSF or blood is thought to reflect the extent and intensity of neuronal damage of any etiology. Oppositely, the presence of post translationally modified tau species, i.e., truncated or phosphorylated, is considered a typical feature of some tauopathies. Blood and CSF levels of tau, but not p-tau, increases after ischemic stroke and mild head trauma, being highly increased in case of prion disease as Creutzfeldt-Jakob disease (114–116). Conversely, in tauopathies such as AD or other dementia, the production of p-tau, C- or N terminal truncated tau species are specifically increased, reflecting underlying neurofibrillary pathology and the formation of post translationally modified species which accumulate in brain and can be also detected in biological fluid (117–119).

Tau proteins have been proposed as candidate markers of stroke and SDNG for evaluating the extent of neurodegeneration, and potentially discriminating neuronal damage with the presence of degenerative processes of other etiology, such as Alzheimer’s pathology. Studies combining CSF analysis and neuroimaging demonstrate a correlation among the magnitude of t-tau increase and the volume of tissue damage following ischemic stroke (81). A recent study compared levels of plasma tau and NfL as cross-sectional and longitudinal markers of cognitive decline and neuroimaging changes, on a cohort of 995 subjects from the community-based Mayo Clinic Study of Aging (MCSA) (82). Both plasma NfL and T-tau have been found to be elevated in stroke patients, and among those with other cardiovascular conditions; the combination of having both higher NfL and T-tau, compared to either alone, was more strongly associated with lower memory, global cognitive decline, brain atrophy and a higher number of infarcts cross-sectionally (82).

Interestingly, in stroke patients, concentration of plasma tau progressively increases from admission reaching a maximum peak 7 days after event, showing a correlation with severity, long-term outcome, as well as infarct volume (83, 84). Analysis of kinetics of tau after global brain ischemia revealed a bimodal elevation after day 2 and 4, probably due to the occurrence of early first necrosis and secondary delayed neuronal death (85, 120). This is also confirmed by a recent retrospective study on head trauma and remote injury, involving a total of 164 subjects, 94 PD patients and 70 healthy controls, in which the levels of CSF tau were found to be increased in a subgroup of PD patients who reported lifetime head trauma preceding diagnosis, probably due to extended degeneration or occurrence of tauopathy (121).

Evidence from experimental models also suggest that tau has a key role in regulating neuronal damage and SDNG after stroke, up to the development of Alzheimer’s-type dementia (64). In animal models, tau protein dysfunction following ischemia may trigger neurofibrillary tangle-like tauopathy and neurofibrillary tangles (86, 87). In humans, history of ischemic stroke increases likelihood of developing AD-type dementia (122) and further, patients suffering of AD with previous brain ischemic injuries show more severe dementia phenotype (123). Certainly, tau in combination with other biomarkers is useful for clinical and research purposes in order to reveal the development of future or clinical subtle neurodegeneration.

### 2.3. S100b

S100b is a calcium-binding protein mainly expressed in astroglial and Schwann cells, myeloid-derived cells, and a few other cell types. Under physiological conditions, S100b is released in the extracellular space in response to hormonal or inflammatory stimuli and exerts both paracrine and autocrine effects on neurons and glia, but increases are observed in neuronal pathological conditions, such as brain trauma, ischemia and neurodegenerative, inflammatory and psychiatric diseases.

S100b is widely used in emergency medicine due to its high positive predictive value in cases of brain injury, showing a correlation with the severity of post-traumatic neurological deficit as well as with the infarct volume after stroke (88–90, 124).

A study on 32 consecutive patients evaluated changes of S100b and the neuron specific enolase (NSE) levels between the first 6 h, and in the 5 days after stroke. Serum concentrations of S100b from 6 h on and of NSE from 24 h on were associated with the outcome at 3 months with a maximum of correlation obtained for protein S100b at 48 h (91).

Moreover, the changes in S100b concentration reflect the extent of astroglial damage, the enlargement of the ischemic core, (92) as well as the formation of malignant edema and of blood–brain barrier damage (93). Conversely, in transient ischemia not associated with substantial tissue injury, S100b levels in serum are generally normal (94, 125). Recently, S100b has been proposed as a surrogate marker to monitor the stroke response after endovascular treatment. Indeed, levels of S100b are correlated with the recanalization following mechanical thrombectomy, being low in case of successful recanalization, and oppositely being increased in case of ineffective recanalization and in patients who developed infarcts despite recanalization (95).

So far, studies in patients with neurodegenerative diseases showed conflicting results. Some authors found that serum S100b concentrations were similar (126) or lower in CSF of AD patients compared with elderly controls, not correlating with brain atrophy (127), whereas others reported correlations between CSF S100b levels and AD brain atrophy or cognitive status as measured by the Mini Mental State Exam score (128, 129).

Notwithstanding, studies are still conducted on limited cohorts, therefore results need to be consolidated by larger cohorts and longitudinal studies, evaluating the association between S100b changes, volume of tissue damage and the functional or cognitive outcome over the post-stroke phases. Although S100B can be considered a useful biomarker in the acute phase after stroke to evaluate the damage of the NVU and astroglial cells, further investigations to understand its role in SDNG following stroke are needed.

### 2.4. Neuron specific enolase

Neuron specific enolase (NSE) is an enzyme involved in glycolytic energy metabolism in the brain, released from neurons during injury as a nonspecific marker of neuronal damage. Studies have investigated the potential of NSE as a predictor of outcome in patients in the early phase after stroke. In 150 cases of patients with stroke and 101 controls, NSE level showed a positive correlation with stroke severity at the time of admission and with the degree of disability, categorized into mild, moderate and severe according to NIHSS; further, NSE was associated with neurological worsening after 7 days of event (96).

Data obtained from 12 studies including 597 patients, found that serum NSE levels were higher in stroke patients compared with controls and correlated with volume of infarct; however, results do not support a correlation among NSE and functional outcome and further the relationship to stroke severity is unclear (97). Increase of NSE as well as of tau was highly correlated with severity of neurological deficits and infarct volume, and with the functional outcome at 3 months. Interestingly, NSE release was associated with the neurovascular status on admission (84).

Findings on CSF in patients with primary neurodegenerative diseases, such as AD, PD, LBD, suggest that NSE may be used to evaluate the presence and extent of axonal and glial degeneration (130). NSE in combination with tau may predict secondary damage after stroke, with specific windows that reflect different release mechanisms (84). In fact, the NSE concentration in serum increase 2–3 h after onset of first stroke, afterwards, NSE decreased, followed by a secondary increase until day 5, that is the last measurement in the observation period; the secondary increase indicates further release of NSE, which probably reflects a secondary mechanism of brain damage, ongoing neuronal cell death (98, 99), or persistent disturbance of the blood–brain barrier (131). A recent study found a correlation between NSE and both stroke severity and prognosis after 1 year of follow up documented by NIHSS score and modified Rankin Scale (mRS) score, respectively, on 1,086 patients grouped as hypertension and non-hypertension (100). However, studies mainly focus on the role of NSE in the acute and subacute phase after stroke and its potential predictive or prognostic value. Longitudinal studies on large cohorts are needed to evaluate the association between NSE levels and the development of brain morphological changes in secondary neurodegeneration.

### 2.5. BDNF

The brain derived neurotrophic factor (BDNF) is the most abundant neurotrophin in the adult brain, and has a remarkable capability to repair brain damage and maintain synaptic plasticity by inducing neuronal proliferation, survival and differentiation (132). However, unlike synaptic plasticity involved in normal cognitive function, post-stroke and rehabilitation neuroplasticity primarily refers to the brain’s ability to recover from injury to restore its normal structure and function. In this context, the significant role of BDNF in the regulation and maintenance of synaptic plasticity after stroke has been extensively investigated in both clinical and experimental studies, including the potential use of BDNF as a direct therapeutic agent for the stroke treatment (133).

After a stroke event, reactive astrocytes upregulate the expression of BDNF and other neurotrophic factors, and the resulting levels have been demonstrated to be associated with the clinical and functional outcome. For instance, BDNF decrease after stroke is associated with poor long term functional outcome (101) and development of post stroke depression (102); further, low level of BDNF is associated with increased risk of stroke (103).

A recent meta-analysis of data from 62 studies showed that subjects with stroke (*n* = 1856) had lower BDNF levels compared to healthy controls (*n* = 1,191), but there was no significant difference in the level of BDNF through time points post stroke. Furthermore, BDNF levels were lower in the patients with depression compared to non-depressed subjects, and positively affected by performing physical training in the early but not in the long term (104).

Preclinical studies demonstrated that BDNF induces anti apoptotic mechanisms, reducing the size of the lesion and secondary death (105), promoting synaptogenesis, neuronal plasticity and recovery post stroke (105, 106); moreover, increase of BDNF concentration in the cortex is related to motor learning after-stroke (107). Experimental studies demonstrated clinically positive outcomes reached by administration of stroke treatments that modulate BDNF expression, leading to consider BDNF as potential therapeutic target (133). Together, these studies suggest that BDNF exerts favorable effects in post-stroke recovery due to its attenuation of cell death and promotion of neurogenesis. However, longitudinal studies investigating the correlation between BDNF levels and other biomarkers of neurodegeneration, as well as morphological and clinical changes, are needed to better understand the development of SDNG and recovery in the chronic phase after stroke.

## 3. Future perspectives

Current therapeutic strategies for post-stroke patients are based on multidisciplinary approaches that include neuropsychological rehabilitation, physical, occupational, speech therapy and neuromodulation techniques, as the recent evidence of effectiveness of Transcranial Direct Current and Transcranial Magnetic Stimulations. Moreover, the prevention of disability must also take into account the presence of a possible secondary brain detriment that is driven by neuroinflammatory cascades and dysfunction of the NVU (39, 134) often leading to the development of SDNG. The early identification of degenerative pathological processes, also not clinically evident, is crucial for the pharmacological and rehabilitative treatment of patients. Neuroimaging techniques, which offer a complete view of the anatomical location and morphological characteristics of pathological processes within the brain, cannot be used as a screening and monitoring tool due to high costs and possible insufficient resolution to detect the early changes in the brain at a cellular and molecular level.

The possibility that biomarkers measured in blood may be predictive of future outcome and association with SDNG is appealing. Several potential markers of neurodegeneration have been identified, which can help capture a range of brain changes and pathologies. Plasma biomarkers, rather than CSF and imaging markers, provide a low-cost, non-invasive tool to evaluate neurodegeneration and to assess rate of disease progression, given the feasibility of repeat blood draws. Notwithstanding, it is crucial to understand what information each blood marker provides to know how they can best be applied for clinical and research purposes (82). Numerous studies have highlighted the correlation between changes in the levels of some biomarkers, such as NfL, tau, and clinical worsening in the chronic phases in stroke patients. Moreover, the levels of biomarkers also correlate with the morphological changes of the brain, suggesting a potential use in clinical settings. However, studies on the significance of changes in biomarker levels in the chronic phase and recovery, or on neurodegenerative mechanisms secondary to stroke, remain elusive and will require further investigation.

Most of the studies have been conducted on limited cohorts of patients and in the hyper, acute or subacute phases. Therefore, larger and longitudinal studies are needed to evaluate the association between biomarkers along the acute and chronic phases after stroke and the functional outcomes, as well as the role of biomarkers in late phase, over a period of years, to evaluate the effect of neuronal degeneration and rehabilitation therapy. For example, the biological efficacy of a rehabilitation method could be monitored by evaluation of specific SDNG biomarkers, e.g., NF-L and BDNF; therefore, in the future, blood biomarkers could be integrated into a tool for defining personalized rehabilitation approaches.

Nevertheless, the interpretation of biomarkers results in a clinical context needs expertise and caution. A limit of biomarkers analysis is that any changes in the levels can reflect acute or progressive pathological brain changes, but do not allow to recognize the etiology or identify where the degeneration is occurring. Therefore, a careful clinical evaluation is always important, and further integration of biochemical markers with neuroimaging is essential to reveal which brain areas are involved.

Moreover, the mechanisms participating in the development of SDNG after stroke have not been fully elucidated. A better understanding of the interlinks between inflammation, oxidative stress and degeneration may help to identify the appropriate biomarkers to be assessed to monitor the therapeutic and rehabilitation treatments. Last, since recent studies suggest that neurogenesis and angiogenesis processes are activated within brain areas after stroke events, it would be interesting to identify novel potential biomarkers associated with regeneration in stroke recovery.

## 4. Conclusion

Stroke care has been revolutionized in the last three decades by improved reperfusion treatments and rehabilitation therapies. The evaluation of neurodegenerative biomarkers in blood shows promising results for clinical and research purposes, especially in the evaluation of acute stroke patients. Further research is needed to better understand the pathophysiology of SDNG after stroke, to develop a useful tool to monitor and detect the occurrence of molecular and cellular pathological changes to finally predict the disability in patients. More rigorous studies should be conducted to validate the potential use of biomarkers in clinical settings, in order to define personalized pharmacological and neurorehabilitative treatments for stroke patients.

## Author contributions

GS, SB, and DD contributed to the conception and design of the manuscript. MB contributed to the figures and neuroimagings. EG contributed to the first draft of the manuscript, the figures, and analysis. All the authors wrote sections of the manuscript and contributed to manuscript revision, read, and approved the submitted version.

## Funding

This study was supported by the Italian Ministry of Health— Ricerca Corrente anno 2023.

## Conflict of interest

The authors declare that the research was conducted in the absence of any commercial or financial relationships that could be construed as a potential conflict of interest.

## Publisher’s note

All claims expressed in this article are solely those of the authors and do not necessarily represent those of their affiliated organizations, or those of the publisher, the editors and the reviewers. Any product that may be evaluated in this article, or claim that may be made by its manufacturer, is not guaranteed or endorsed by the publisher.
